# Mapping and Quality Appraisal of Artificial Intelligence Preferential Reporting Checklists, Items, Guidelines, and Consensus in Healthcare: An Altmetric, Bibliometric, and Systematic Review

**DOI:** 10.1155/ijod/6730710

**Published:** 2026-04-18

**Authors:** Vineet Vinay, Praveen Jodalli, Mahesh S. Chavan, Dharmashree Satyarup, Ketaki Bhor, Chaitanya Buddhikot

**Affiliations:** ^1^ Department of Public Health Dentistry, Manipal College of Dental Sciences Mangalore, Manipal Academy of Higher Education, Manipal, India, manipal.edu; ^2^ Department of Oral Medicine and Radiology, Sinhgad Dental College and Hospital, Pune, Maharashtra, India, sdchpune.org; ^3^ Department of Public Health Dentistry, Institute of Dental Sciences, Siksha O Anusandhan Deemed to be University, Bhubaneshwar, Odisha, India, soauniversity.ac.in; ^4^ Department of Public Health Dentistry, Government Dental College and Hospital, Mumbai, Maharashtra, India, gdcmumbai.org; ^5^ Department of Public Health Dentistry, Dr. D. Y. Patil Dental College and Hospital, Dr. D. Y. Patil Vidyapeeth (Deemed to be University), Pimpri, Pune, Maharashtra, India, dpu.edu.in

**Keywords:** artificial intelligence, checklist, guidelines, healthcare, reporting standards

## Abstract

**Introduction:**

The integration of artificial intelligence (AI) in healthcare has garnered significant scholarly attention, particularly in areas such as medical image analysis, prognosis, and treatment. Despite its potential, concerns regarding AI’s reliability and application persist, prompting the development of guidelines aimed at standardizing its use in medicine. This study aims to evaluate the current content and quality of AI guidelines in healthcare, focusing on identifying gaps and providing a critical appraisal of existing checklists.

**Methodology:**

Comprehensive bibliometric analysis, Altmetric analysis, and systematic review were conducted, utilizing the AGREE II Tool for quality appraisal. The systematic search spanned Scopus, PubMed, and Dimension AI databases, focusing on English‐language, open‐access articles related to AI reporting guidelines. Two reviewers independently evaluated the data, with manual extraction performed in Microsoft Excel. The AGREE II Tool assessed six domains of guideline quality.

**Results:**

The search yielded 2477 articles, ultimately identifying 27 AI‐specific reporting guidelines published between 2020 and 2025. The analysis revealed significant variations in quality across the AGREE II domains. Among the very first and most impactful were SPIRIT‐AI, CONSORT‐AI, MINIMAR, and CLAIM, all prioritized structured reporting but were hampered by their timing—based when there were few AI trials—resulting in limited applicability and risk of missing older AI terminologies. However, STAR‐machine learning (ML), APPRAISE‐AI, and CLEAR exhibited more general, domain‐specific frameworks, whereas checklists such as CHEERS‐AI, CREMLS, and MI‐CLEAR‐large language model (LLM) showcased limited author diversity in contribution. However, many guidelines exhibited weaknesses in methodological rigor and stakeholder involvement, limiting their practical applicability.

**Conclusion:**

The findings emphasize the need for evidence‐based updates to AI reporting guidelines to ensure methodological integrity amid rapid advancements. Increased expert involvement and stakeholder engagement are crucial for enhancing the guidelines’ applicability and rigor, addressing AI complexities in health research, and adapting reporting frameworks to evolving AI technologies in healthcare.

## 1. Introduction

Over the course of a few years, a significant amount of attention has been paid to artificial intelligence (AI) in the field of healthcare practices. AI has become a transformative force in modern diagnostics, clinical decision‐making, workflow efficiency, and patient monitoring. Advances in machine learning (ML), deep learning, and more recently large‐scale generative models are enabling systems with unprecedented capacities for pattern recognition and automated inference. Foundational reviews have underscored the broad range of AI applications across imaging, predictive analytics, and biomedical data interpretation, reflecting rapid methodological progress and increasing clinical integration [1, 2]. This expansion has been accompanied by exponential growth in global AI‐related research output, as demonstrated through multiple bibliometric analyses that document rising publication volume, diversification of research themes, and widening international participation in health‐related AI scholarship [3–5]. Applications of AI in the medical field include medical image analysis and text mining, with a particular emphasis on prognosis, diagnosis, making predictions about events and outcomes, and treatment [1]. There has been a significant increase in the number of scientific contributions that are being published in journals that are subject to peer review and that are concerned with the uses of AI in medicine [4, 5].

Despite this global surge, concerns persist regarding bias, reproducibility, safety, and the real‐world reliability of AI systems. Structural challenges—such as biased datasets, opaque models, and inadequate evaluation methodologies—continue to limit the safe translation of AI from the laboratory setting into clinical practice [6]. Moreover, susceptibility to adversarial attacks raises safety questions for even the most advanced models. Studies have shown that subtle perturbations in medical images can cause AI systems to produce incorrect or potentially harmful predictions, emphasizing the importance of robust evaluation and transparent documentation of model development and validation [7]. These risks are further compounded by the emergence of large language models (LLMs) and automated text‐generation systems, which can mislead through fabricated content when deployed in clinical or scientific contexts [8, 9].

In recognition of these challenges, the scientific and regulatory communities have emphasized the necessity of comprehensive reporting frameworks. There have been guidelines, consensus statements, and standards produced to facilitate the increase in the standardization of the application of AI in medicine. These guidelines and standards include suggestions that are meant to improve patient care and make use of data from systematic reviews and evaluations of possible benefits and risks [10]. When it comes to comprehending and putting into action ideas that improve applications in medicine, guidelines that are clear, precise, and transparent can be of great assistance to healthcare practitioners, administrators, program managers, and the public [10, 11].

As the clinical use of AI systems continues to improve, it will become increasingly important to adhere to AI guidelines and to update them on a regular basis [12]. Guidelines on AI medicine that are of a high quality would be of use to professionals in improving decision‐making and incorporating the most compelling evidence into AI systems. Quality problems that are caused by the application of AI continue to exist [13, 14].

The consequences of suboptimal or inconsistent reporting extend beyond the research community. Inadequate use or selective adoption of guidelines can jeopardize clinical implementation, mislead policymakers, and impede regulatory evaluation of AI interventions [14]. Evidence from other fields underscores the importance of guideline quality and accessibility.

The current content and quality of AI guidelines in the field of healthcare research need to be evaluated to determine the scope of these problems, which include medical research, medical practice, and other applications. Hence, the present objectives of this study were to identify the overall attention score, scope the bibliometric literature and provide its critical quality appraisal, and identify the lacunae of the checklist published dwelling AI.

## 2. Materials and Methods

### 2.1. Protocol and Registration

A comprehensive bibliometric analysis, Altmetric analysis, and systematic review was conducted for the study purpose. The quality of the guidelines for systematic review was critically appraised using the AGREE II Tool [15]. The data for bibliometric analysis was evaluated using VOS Viewer Version 1.6.19. The systematic review was conducted following Preferred Reporting Items for Systematic Review and Meta analysis 2020 guidelines [16]. The review was prospectively registered in PROSPERO under the Registration Number: CRD420251064187.

### 2.2. Review Question

What is the current content and quality of existing AI guidelines in healthcare, and what gaps or limitations can be identified through a critical appraisal of these checklists?

### 2.3. Eligibility Criteria

All the available guidelines, checklists and reporting items on the topic for reporting AI research in the field of healthcare were identified. Studies were strictly restricted to the English language and open access free full text articles were only selected for the study purpose, which strictly ensured rigorous methodological appraisal, reproducibility, and accurate data for bibliometric and Altmetric analysis (Table 1).

**Table 1:** Inclusion and exclusion criteria.

Inclusion criteria	Exclusion criteria
1. All available guidelines, checklists, and reporting items on topic for reporting artificial intelligence 2. Articles in the English language 3. Articles which are open access 4. Articles that are free full‐text	1. Articles in languages other than English 2. Studies describing only the application of AI (without introducing or evaluating a reporting checklist/guideline) 3. Narrative reviews or editorials without a systematic methodology

### 2.4. Data Sources and Search Strategy

A systematic search was conducted in Scopus, PubMed, and Dimension AI databases from January 2020 till December 2025. The search strategy was formulated by two authors, Vineet Vinay and Praveen Jodalli. The third author, Praveen Jodalli, verified the terms and rectified search terms. Vineet Vinay searched the articles on the databases mentioned above and conducted the extraction. Rayyan 1.4.3 software was used for systematically segregating removal of duplicates and selection of articles.

### 2.5. Scopus

(TITLE‐ABS‐KEY ((“reporting guideline ^∗^” OR “reporting checklist ^∗^” OR “reporting item ^∗^” OR “reporting standard ^∗^” OR “reporting statement ^∗^” OR “consensus statement” OR “checklist” OR “framework”)) AND TITLE‐ABS‐KEY ((“artificial intelligence” OR “AI” OR “machine learning” OR “deep learning” OR “neural network ^∗^” OR “large language model ^∗^” OR “LLM” OR “generative AI” OR “chatbot ^∗^” OR “transformer model ^∗^”))) AND (LIMIT‐TO ( DOCTYPE, “ar”) OR LIMIT‐TO ( DOCTYPE, “re”)) AND (LIMIT‐TO ( LANGUAGE, “English”)) AND (LIMIT‐TO (OA, “all”)).

### 2.6. PubMed

((“reporting guideline ^∗^”[Title/Abstract] OR “reporting checklist ^∗^”[Title/Abstract] OR “reporting item ^∗^”[Title/Abstract] OR “reporting standard ^∗^”[Title/Abstract] OR “reporting statement ^∗^”[Title/Abstract] OR “consensus statement”[Title/Abstract] OR “checklist”[Title/Abstract] OR “framework”[Title/Abstract]) AND (“artificial intelligence”[Title/Abstract] OR “AI”[Title/Abstract] OR “machine learning”[Title/Abstract] OR “deep learning”[Title/Abstract] OR “neural network ^∗^”[Title/Abstract] OR “large language model ^∗^”[Title/Abstract] OR “LLM”[Title/Abstract] OR “generative AI”[Title/Abstract] OR “chatbot ^∗^”[Title/Abstract] OR “transformer model ^∗^”[Title/Abstract])) AND (“loattrfree full text”[Filter]) AND(english[Language]).

### 2.7. Dimension AI

((title_abstract_all = (“reporting guideline ^∗^” OR “reporting checklist ^∗^” OR “reporting item ^∗^” OR “reporting standard ^∗^” OR “reporting statement ^∗^” OR “consensus statement” OR “checklist” OR “framework”) AND title_abstract_all = (“artificial intelligence” OR “AI” OR “machine learning” OR “deep learning” OR “neural network ^∗^” OR “large language model ^∗^” OR “LLM” OR “generative AI” OR “chatbot ^∗^” OR “transformer model ^∗^”) AND language = “English ”AND open_access = true AND year > = 2019)).

### 2.8. Study Selection and Enrollment and Data Extraction

Two reviewers, Vineet Vinay and Praveen Jodalli, independently evaluated the data for titles and abstracts for eligibility and performed data extraction. The interviewer agreement was evaluated using Cohen’s kappa statistics; an agreement of 0.80 (almost perfect) was only selected in the study; however, in terms of discrepancies, an author, Chaitanya Buddhikot, intervened in response to any queries pertaining to the selection of the articles. The data extraction was performed manually in Microsoft Excel Version 13.0. (Table 2). Data collection was done under the categories of year of publication, country, reporting domain, limitations of checklist, and Altmetric Attention Score (AAS).

**Table 2:** Data extraction.

Sr. No.	Authors	Checklist name	Year of publication	Country	Reporting domain	Limitations of checklist
1	Cruz Rivera [17]	SPIRIT‐AI	2020	UK	Guideline	First, it was created in a context where only a few AI trials had been published, which may limit its applicability to real‐world examples. Second, the literature search for relevant trials did not include older terminologies related to AI, potentially overlooking significant studies. Last, the initial candidate items were generated by a small group of experts, although broader input was sought through the Delphi process. As AI research evolves, ongoing collaboration with investigators is encouraged to ensure the relevance of these reporting standards
2	Liu et al. [18]	CONSORT‐AI	2020	UK	Guideline	The literature search focused on modern AI terms, potentially overlooking earlier systems like clinical decision support systems that share similar risks. The checklist serves as a minimum standard, and additional AI‐specific considerations may be necessary for comprehensive reporting. AI technology is rapidly changing, necessitating ongoing updates to the checklist to remain relevant as new applications and challenges arise
3	Mongan et al. [19]	CLAIM	2020	USA	Checklist	Limitations of the checklist have not been clearly stated
4	Olczak [20]	Clinical AI Research (CAIR) checklist	2020	Sweden	Checklist	Sensitivity and specificity have been calculated; however, procedure for updating the guideline is not included
5	Hernandez‐Boussard et al. [21]	MINIMAR	2020	USA	Reporting Standards and Checklist	Strengths and limitations not mentioned, future updating protocols not mentioned
6	Vasey [22]	DECIDE‐AI	2022	UK	Reporting guideline	The recommendations are not thoroughly specific
7	Daneshjou [23]	CLEAR Derm Consensus Guidelines	2022	USA	Checklist	The authors have failed to give a procedure for updating the guideline
8	Koh [24]	STAR‐ML	2023	Canada	Checklist	Not mentioned
9	Kwong et al. [25]	APPRAISE AI	2023	Canada	Checklist	The checklist is not externally reviewed by experts prior to its publication
10	Kocak [26]	CLEAR	2024	Turkey	Guideline	Limitations include reliance on a single citation source a limited number of articles analyzed, and potential biases due to the authors’ involvement in the guideline’s development
11	Elvidge [27]	CHEERS‐AI	2024	UK	Guideline	Despite its comprehensive nature, CHEERS‐AI has limitations. The Delphi study used to develop it involved a smaller group of experts compared to other similar initiatives, which may have restricted the diversity of insights. Additionally, while the checklist aims to enhance reporting quality, pilot testing revealed that many EEs still lack adequate reporting on AI‐specific items, indicating ongoing gaps in the evidence base rather than flaws in the checklist itself
12	Collins [28]	TRIPOD‐AI + PROBAST‐AI	2024	UK	Guidelines	The different options for management of the condition or health issue are not clearly presented
13	El Emam et al. [29]	CREMLS	2024	Canada	Reporting guideline	The authors are thorough with the guidelines; however, future recommendations are not elaborated upon
14	Park et al. [30]	MI‐CLEAR‐LLM	2024	Korea	Reporting guideline	All the authors of the checklist are from a single field, which may give rise to bias
15	Kameyama et al. [31]	ELSI	2024	Japan	Reporting guideline	Authors have not systematically mentioned the methods used to search for evidence
16	Sallam et al. [32]	METRICS	2024	Jordan	Checklist	The authors have given the screening procedure in detail; however, view and preferences of the target population are not mentioned
17	Chen [33]	STAGER checklist	2024	China	Checklist	Funding for the study is not mentioned by the authors, future recommendations, limitations, and strength of the study are not provided
18	Huo [34]	CHART	2025	Canada	Guideline	Rapidly evolving study designs may limit its applicability, while CHART aims to improve the quality, it does not serve as appraisal tool for the literature
19	Brankovic [35]	CLIX‐M	2025	Australia	Checklist	Lack of standardized metrics, clinician‐informed XAI evaluation checklist with metrics (CLIX‐M) for AI‐powered clinical decision support systems, clinician‐informed XAI evaluation XAI checklist with metrics (CLIX‐M) for AI‐powered clinical decision support systems
20	Anibal [36]	i CARE	2025	USA	Reporting Standards and Checklist	While the checklist aims to enhance transparency and robustness in AI research, it may not address the inherent challenges of AI, such as data biases, algorithmic limitations, and the generalizability of models across diverse patient populations. The reliance on self‐reported data from research could also introduce biases or inaccuracies in the information provided. Last, the checklist does not guarantee that the AI models will be effectively deployed in clinical settings, as many AI innovations fail to transition from research to real‐world applications
21	Kwak and Kim [37]	Statistical Round of the Korean Journal of Anesthesiology	2025	Korea	Reporting guideline	The guidelines were developed solely by two experts in statistics, which may not encompass a broad range of insights from other AI specialists. The discussion emphasizes the need to address human errors in data preparation, external factors affecting AI performance, and ethical considerations regarding equity in AI model development
22	Yang [38]	Clin‐STAR	2025	USA	Extension checklist	Around the EQUATOR With Clin‐STAR: AI‐Based Randomized Controlled Trial Challenges and Opportunities in Aging Research
23	Gallifant [39]	TRIPOD‐LLM	2025	USA	Reporting guideline	It does not prescribe how to develop or evaluate LLMs, nor does it serve as a quality appraisal tool. Additionally, it is designed specifically for text‐based LLMs, and while it may evolve to include multimodal models, unique considerations for such models are not yet fully addressed
24	Sounderajah [40]	STARD‐AI	2025	UK	Reporting guideline	Limitations of the checklist include its length, which has increased due to the addition of AI‐specific items, potentially posing a barrier to implementation. Furthermore, the rapid advancement of AI technology may outpace the checklist’s relevance, necessitating regular updates to address new developments in AI models and their applications in clinical settings. Additionally, while STARD‐AI focuses on diagnostic accuracy, it does not provide detailed instructions for authors, which may limit its utility in ensuring comprehensive reporting. Last, the guideline’s effectiveness in enhancing transparency and reproducibility in AI diagnostic studies depends on its adoption by researchers, journals, and regulatory bodies
25	Luo [41]	GAMER	2025	Singapore	Guideline	First, it does not address non‐GAI tools or general web search engines, which may limit its applicability in broader contexts. Additionally, while the checklist aims to improve reproducibility and trustworthiness, the effectiveness of its implementation relies on widespread adoption by journals and researchers, which may vary. Last, the checklist may not cover all unique challenges posed by GAI tools, as technology continues to evolve rapidly, potentially outpacing the guidelines established
26	Moons [42]	PROBAST+AI	2025	Netherlands	Reporting guideline	The different options for management of the condition or health issue are not clearly presented
27	Fleurence [43]	ELEVATE‐GenAI	2025	USA	Reporting guideline	The views and preferences of the target population are not included

### 2.9. Quality and Risk of Bias

The quality of the guidelines was appraised systematically using the AGREE II Tool as mentioned above. The AGREE II Tool consists of six Domains with 23 questions, following questions pertaining to rating the overall quality of the guidelines and recommendation. The six domains were evaluated by four authors independently as per the requirement of the AGREE II Tool for its final appraisal. The domains evaluated were scope and purpose, stakeholder involvement, clarity of presentation, applicability, and editorial independence.

The final score was obtained and computed for each domain using the formula provided by the authors of the AGREE II guideline, that is, domain score = ([obtained score – minimum score]/[maximum score – minimum score]) x 100 for obtaining the final percentage of the domain score (Table 3).

**Table 3:** Altmetric attention and AGREE II guidelines evaluations.

Sr. No.	Checklist name	Altmetric Attention Score	D1	D2	D3	D4	D5	D6
1	SPIRIT‐AI	118	97.22	100.00	97.92	100.00	100.00	100.00
2	CONSORT‐AI	288	100.00	100.00	97.40	100.00	100.00	100.00
3	CLAIM	3	100.00	33.33	29.17	48.61	62.50	62.50
4	Clinical AI Research (CAIR) checklist	3	81.94	43.06	42.71	50.00	75.00	37.50
5	MINIMAR	23	65.28	50.00	18.23	34.72	27.78	76.39
6	DECIDE‐AI	108	100.00	100.00	100.00	100.00	100.00	69.44
7	CLEAR Derm Consensus Guidelines	40	100.00	75.00	73.44	77.78	62.50	76.39
8	STAR‐ML	4	88.89	70.83	50.52	75.00	86.11	37.50
9	APPRAISE AI	15	65.28	77.78	88.54	34.72	70.83	88.89
10	CLEAR	1	100.00	94.44	98.96	100.00	100.00	100.00
11	CHEERS‐AI	31	100.00	100.00	100.00	100.00	100.00	100.00
12	TRIPOD‐AI + PROBAST‐AI	146	88.89	87.50	98.96	100.00	100.00	100.00
13	CREMLS	15	97.22	100.00	100.00	100.00	100.00	100.00
14	MI‐CLEAR‐LLM	0	73.61	37.50	39.06	26.39	45.83	65.28
15	ELSI	1	80.56	50.00	25.00	50.00	50.00	38.89
16	METRICS	Not mentioned	81.94	73.61	69.79	29.17	62.50	76.39
17	STAGER checklist	2	81.94	58.33	41.15	38.89	40.28	0.00
18	CHART	37	97.22	97.22	89.06	81.94	100.00	97.22
19	CLIX‐M	2	98.61	76.39	81.77	70.83	100.00	88.89
20	iCARE	6	93.06	65.28	25.52	25.00	76.39	100.00
21	Statistical Round of the Korean Journal of Anesthesiology	1	100.00	56.94	78.65	81.94	100.00	88.89
22	Clin‐STAR	4	100.00	58.33	95.83	91.67	100.00	100.00
23	TRIPOD‐LLM	53	97.22	100.00	97.92	100.00	100.00	66.67
24	STARD‐AI	33	100.00	100.00	98.96	100.00	100.00	66.67
25	GAMER	12	100.00	97.22	97.92	100.00	100.00	100.00
26	PROBAST+AI	81	100.00	100.00	97.92	100.00	100.00	100.00
27	ELEVATE‐GenAI	9	72.22	54.17	68.23	50.00	70.83	66.67

Interrater reliability was done for four authors who evaluated the Agree II Tool using IBM SPSS Version 21.

### 2.10. Bibliometric Analysis

The bibliometric analysis followed a stringent search strategy wherein a group of authors decided thekey search terms that were to be searched in the database.

As an overall depiction and visualization of the studies published was intended, we included all the articles that were present.

The analysis of the bibliometric content was done using VOS Viewer Version 1.6.18. The analysis included visualization of the data for co‐authors for assessing the authors’ citations, organizations having co‐authors, countries, establishment of the co‐occurrences of the author keywords, obtaining the citations between sources and authors, and bibliographic coupling between the documented sources. For keyword and title term co‐occurrence mapping, a minimum threshold of three occurrences was used to ensure the identification of meaningful themes. Full counting was used in all analyses to give equal weight to all occurrences or co‐authorship. Association strength normalization was employed to account for variations in publication and citation counts between items, thus enhancing comparability in the network. The clustering resolution parameter was set to the default value of 1.00 to prevent the artificial inflation or fragmentation of clusters. The default layout and attraction‐repulsion parameters were also used to prevent human bias in the spatial layout. Before analysis, data cleaning steps were undertaken, including the merging of synonymous terms (e.g., “CONSORT AI” and “CONSORT‐AI”), the standardization of author and institutional names, and the removal of generic, noninformative terms. These steps ensured a systematic, reproducible, and interpretable bibliometric mapping of AI reporting guideline literature.

**Figure 1:**
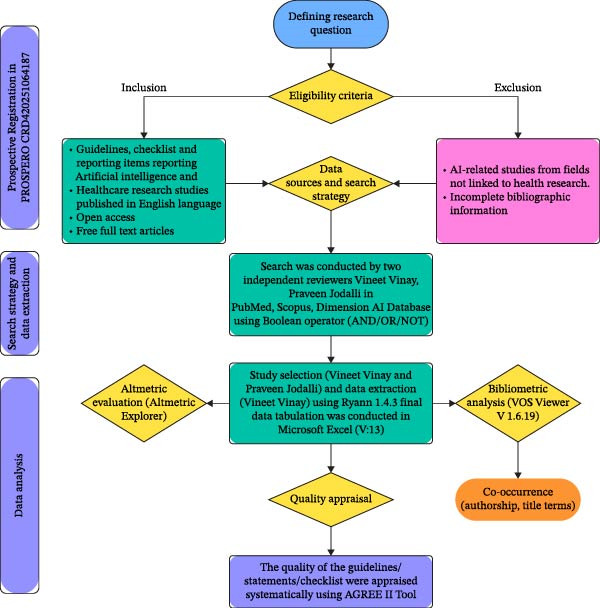
Methodology flowchart.

The summary of methodology is given in the flowchart in Figure 1.

## 3. Results

**Figure 2:**
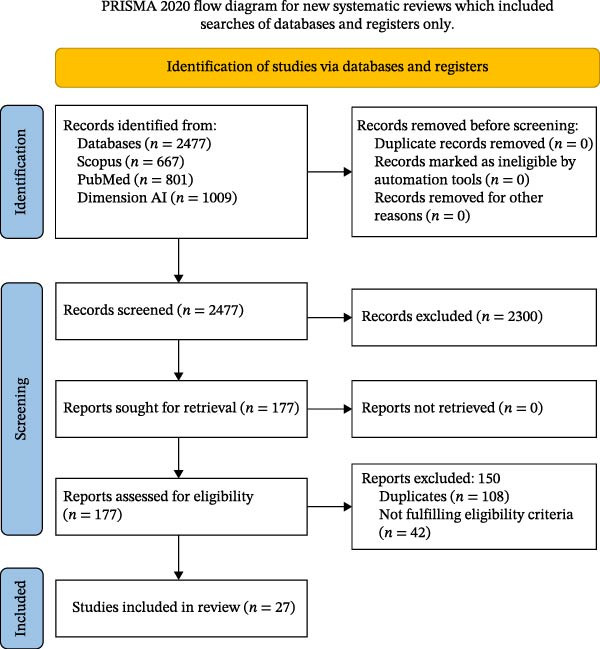
PRISMA flowchart.

The initial search revealed a total of 2477 articles through Scopus, PubMed, and Dimension AI databases. Additionally, two studies were included using a manual search in the Equator Network. The two reviewers, Vineet Vinay and Praveen Jodalli, independently screened and extracted the data for its final evaluation. Finally, a total of 27 guidelines were included for systematic review purposes (Figure 2).

The selection of the articles for the systematic review has been depicted in Figure 1, the PRISMA 2020 Flow diagram.

### 3.1. Bibliometric Findings

#### 3.1.1. Scopus

Scopus data evaluation revealed that of the total 33 countries, 23 had link strengths for co‐authorship analysis. Further, it was observed through overlay visualization that India, Norway, Greece Slovenia, and Spain had co‐authorship post‐2023 time period depicting a rise in country‐wise co‐authored literature.

**Figure 3:**
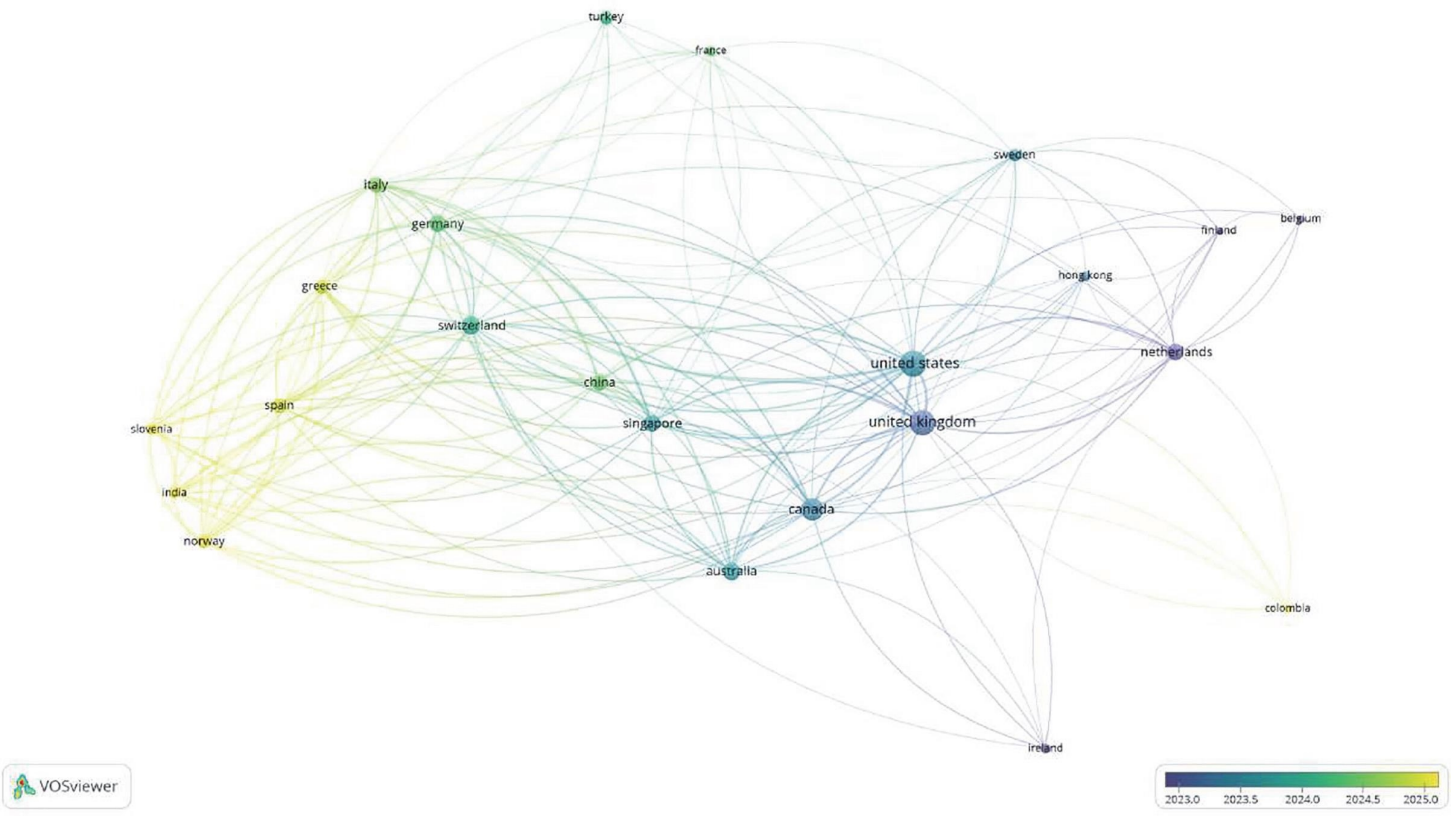
Co‐authorship countries (Scopus).

**Figure 4:**
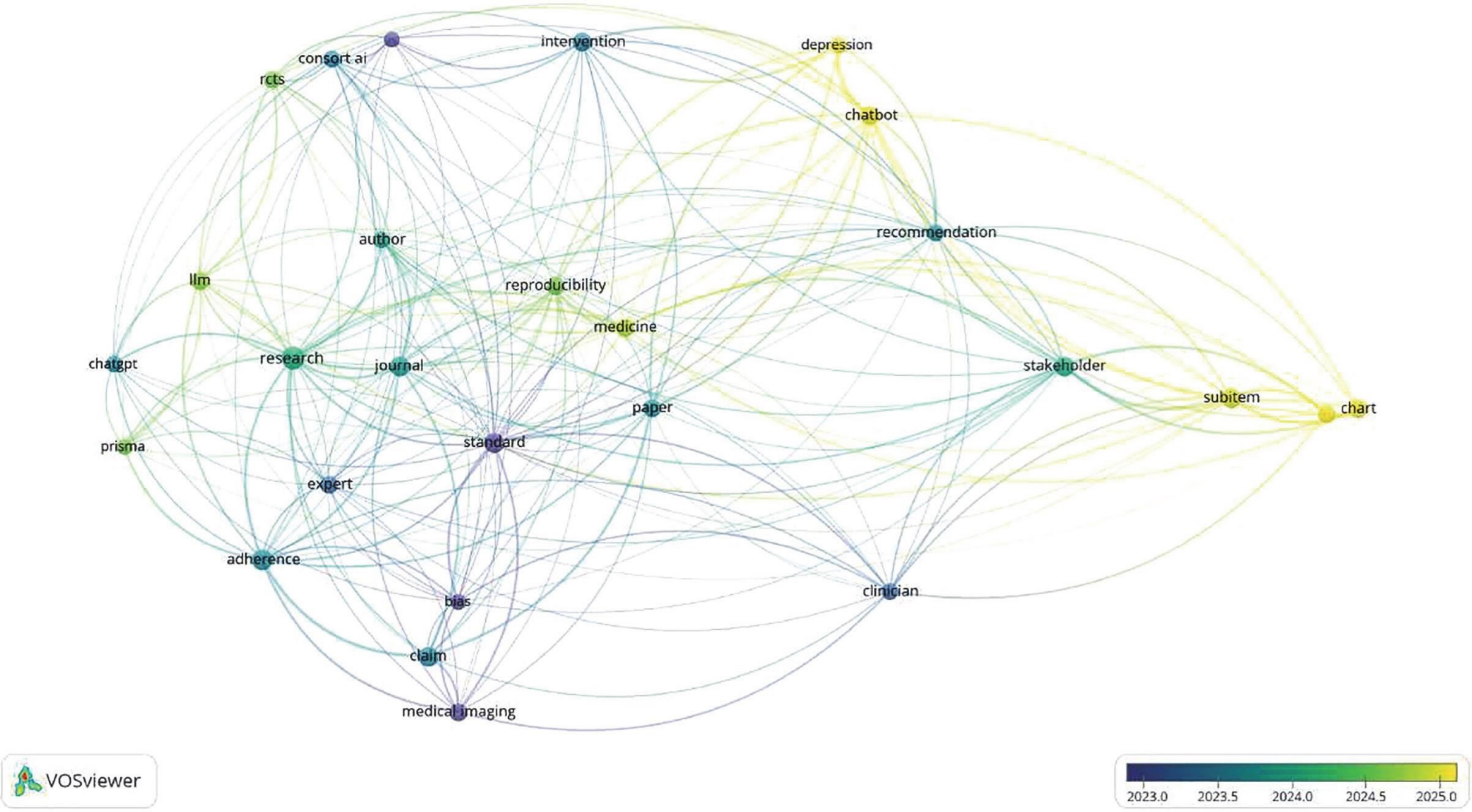
Co‐occurrence of title terms Scopus.

The co‐occurrences on map‐based text data identified that while previous publications pertaining to AI in RCT were evaluated using CONSORT‐AI, post‐2024, CHART‐AI Checklist has depicted a rise as the studies pertaining to ChatGPT have shown a significant increase in literature. A strong network was observed to be associated with authors recommending the usage of checklists in the study designs involving AI (Figures 3 and 4).

### 3.2. Dimensions AI Database

**Figure 5:**
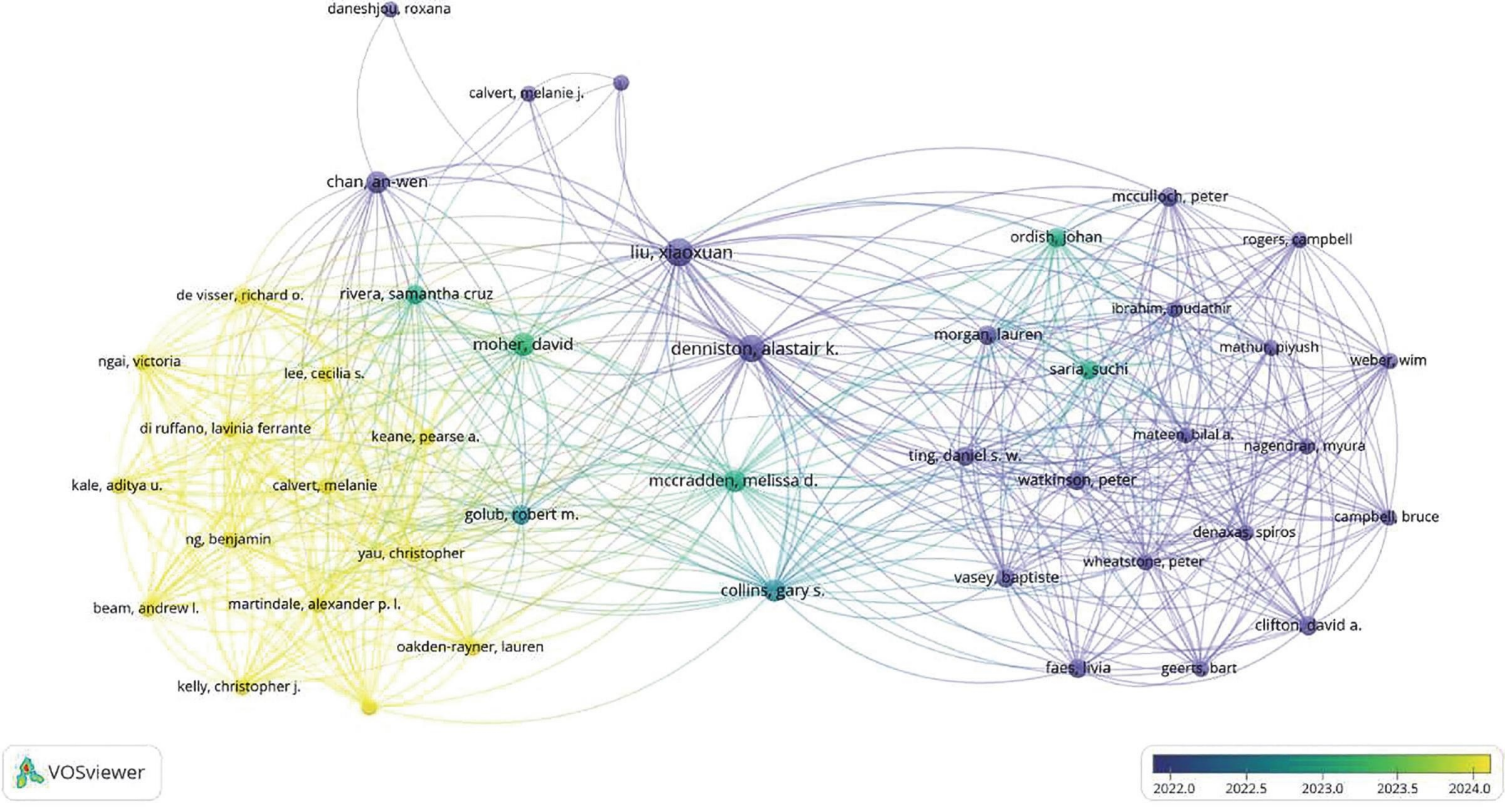
Co‐occurrence of authors Dimension database.

**Figure 6:**
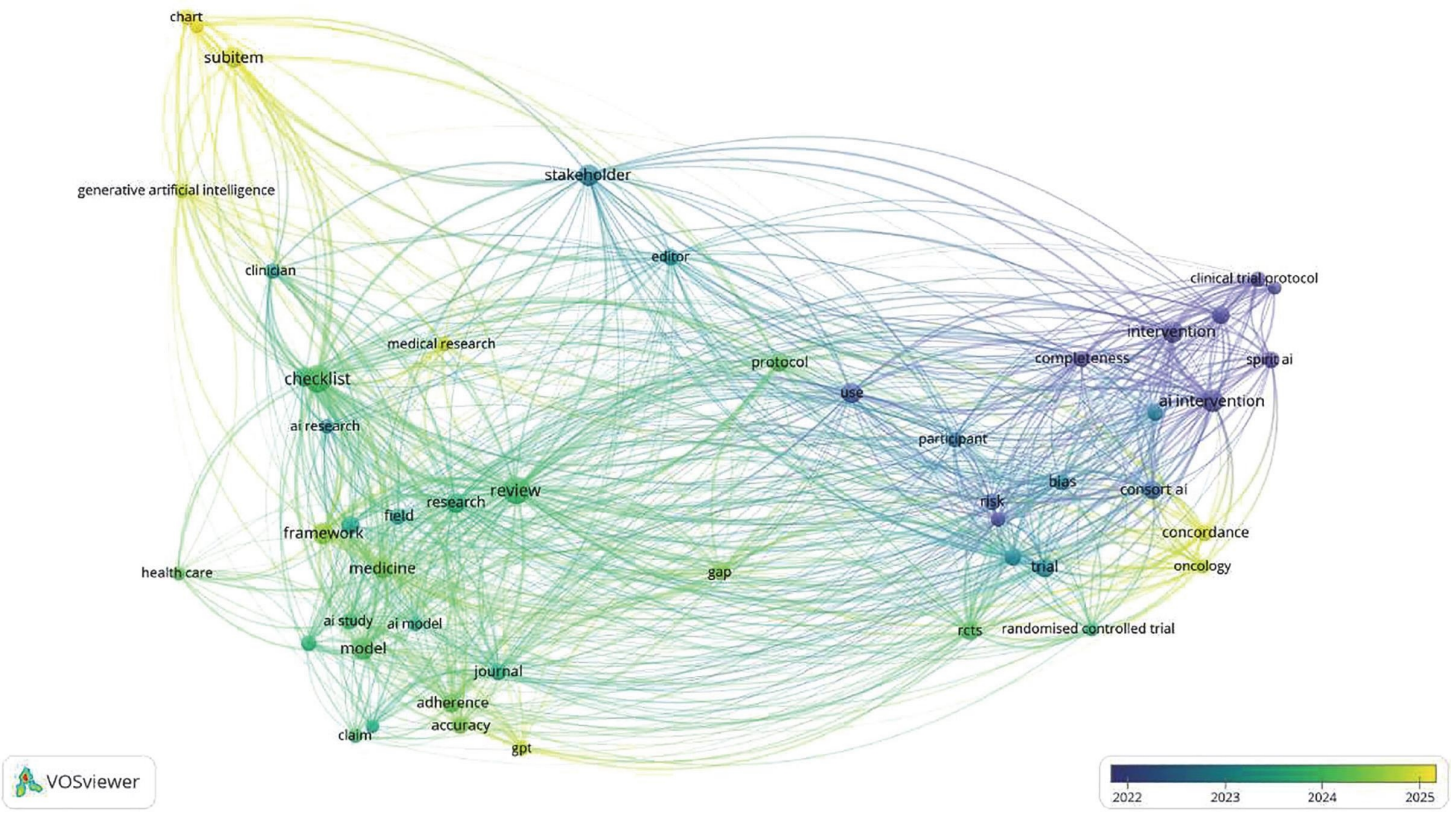
Co‐occurrence of title terms Dimension database.

The co‐authorship analysis was explored using the Dimensions‐based data, which revealed 406 authored literatures items, of which 62 met the threshold of two documents per author and two citations for authors. It was observed that an article published by Liu, Xioxuan had 11 documents with 1322 citations with 112 total link strength. This literature was observed to be published in 2020 pertaining to reporting guidelines for clinical trials reports for interventions involving AI. This literature depicted having an RCR of 12.32 and FCR of 73.16. The literature was observed to be cited 277 times. Another finding noted was pertaining to the title “Reporting guidelines for clinical trial reports for interventions involving AI: the CONSORT‐AI extension” by Chan, An‐Wen; Darzi, Ara et al., who were cited 648 times with 289 recent time citations and 27.01, 177.71 RCR and FCR ratio. The co‐occurrence of the title words depicted a similar finding related to recent time publications using the CHART Statement. As per the visualization, it was observed that researchers appraised using CONSORT‐AI checklist; however, the recent increase in usage of the chatbots has structurally influenced the trend of appraisal using the CHART Statement (Figures 5 and 6).

### 3.3. PubMed Database

**Figure 7:**
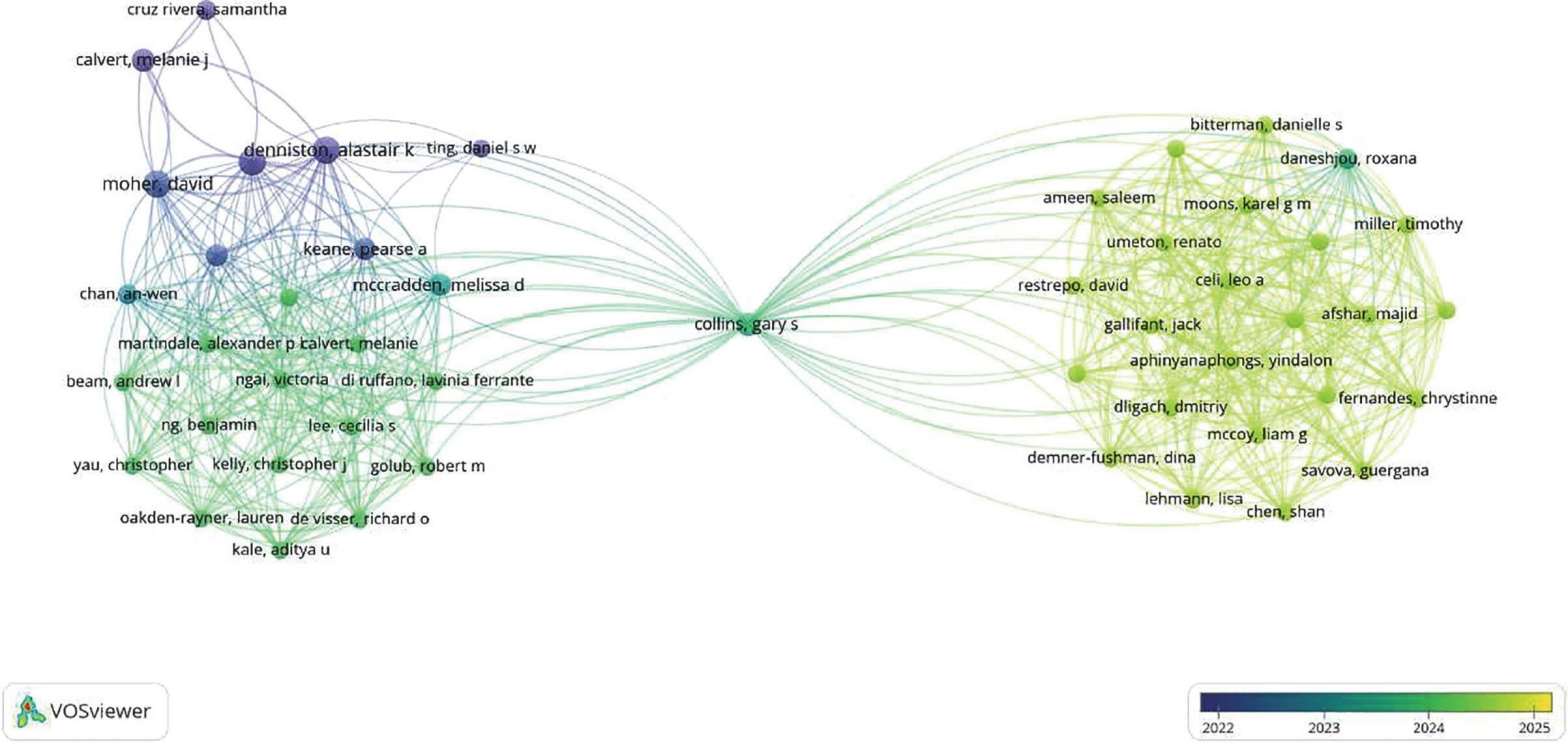
Co‐occurrence authors PubMed.

**Figure 8:**
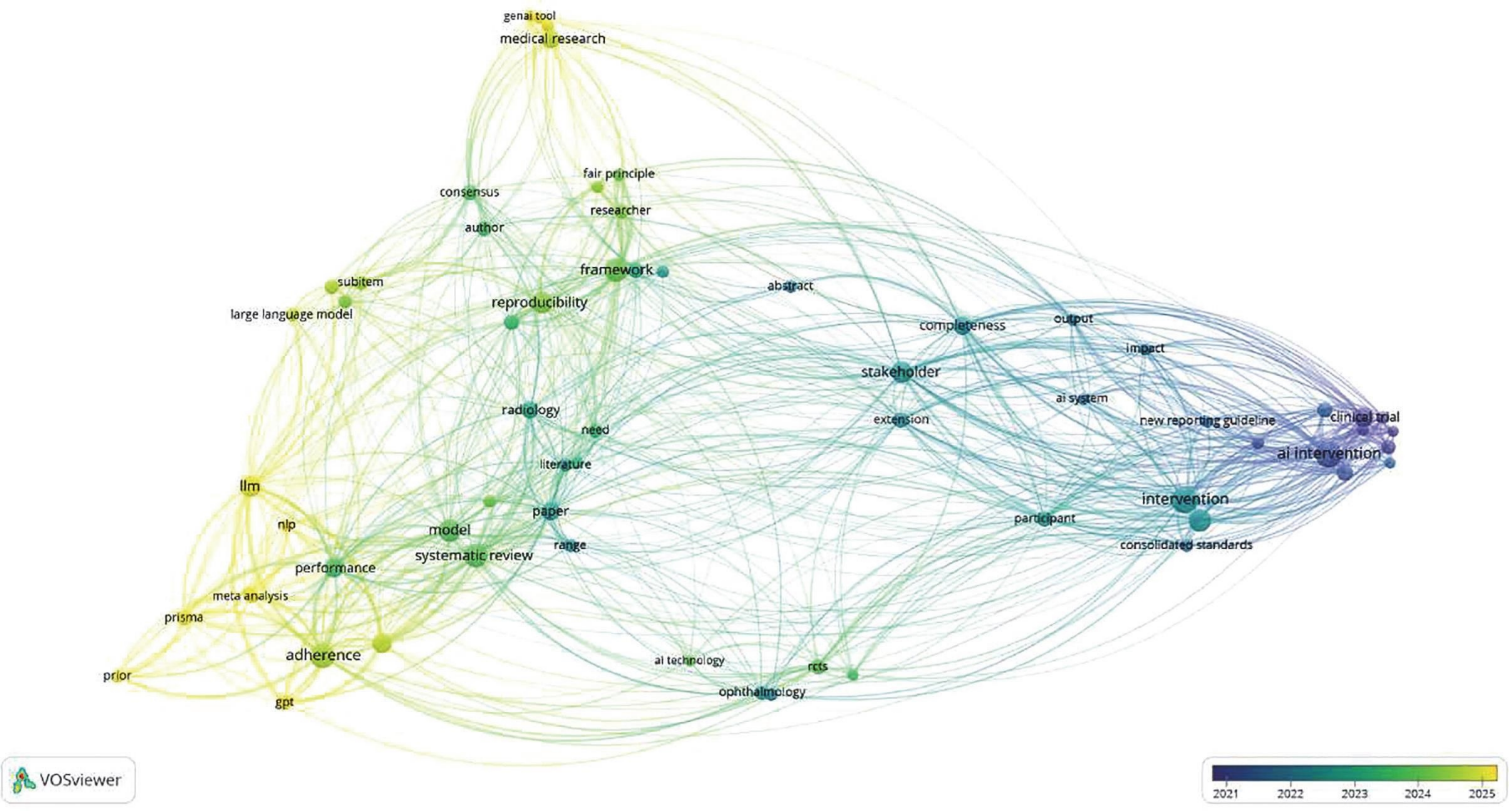
Co‐occurrence of title terms PubMed database.

The data visualization depicted that Collins Gray had significantly impacted the co‐authorship analysis through his literature, “The TRIPOD‐LLM reporting guideline for studies using large language models.” The co‐occurrence of the terms depicted that the GAMER Statement was linked recently to multiple articles and had 6 occurrences with 114 link strengths, while a similar trend was observed toward co‐occurrence of CONSORT‐AI (Figures 7 and 8).

Twenty‐seven AI‐specific reporting guidelines and checklists published from 2020 to 2025 were identified and critically assessed in this systematic review. These checklists were constructed in various countries, such as the UK, USA, Canada, Sweden, Turkey, Japan, China, Jordan, Korea, Australia, the Netherlands, and Singapore, as a reflection of international interest in improving transparency, reproducibility, and quality in AI‐derived health research. The reporting instruments differed in scope, ranging from guidelines, checklists, reporting standards, and extension checklists, and addressed the range of reporting areas, such as diagnostic accuracy, prediction modeling, clinical trial reporting, and ethical use of AI.

Among the very first and most impactful were SPIRIT‐AI and CONSORT‐AI [17, 18] (UK), which adapted prevalent clinical trial frameworks to include AI interventions. Both prioritized structured reporting but were hampered by their timing—based on a period when there were few AI trials—resulting in limited applicability and risk of missing older AI terminologies. The same issues occurred with MINIMAR [21], (USA) and CLAIM [19], (USA), wherein explicit policies for updates and strength–limitation analyses were not defined. Follow‐up projects like DECIDE‐AI [22], (UK) and CLEAR Derm [23], (USA) were centered on practical AI applications but were criticized for a lack of procedural transparency and low specificity.

The introduction of more general, domain‐specific frameworks like STAR‐ML [24], (Canada), APPRAISE‐AI [25], (Canada), and CLEAR [26] (Turkey), with the latter also recognizing possible bias in reliance on single sources of data. CHEERS‐AI [27] (UK) guidelines were found to be limited by a smaller Delphi panel of experts, possibly limiting diversity in contribution. CREMLS [29], (Canada) and MI‐CLEAR‐LLM [30], (Korea) aided in data sharing and LLM reporting but were also at risk of bias with author diversity being low.

New tools of 2025, such as CHART [34], (Canada), CLIX‐M [35], (Australia), iCARE [36], (USA), Clin‐STAR [38], (USA), TRIPOD‐LLM [39], (USA), STARD‐AI [40], (UK), GAMER ([41], Singapore), and ELEVATE‐GenAI [43], (USA), are an evolution toward dealing with the issues of generative AI, explainable AI, and multimodal models. Nevertheless, these tools also had common ongoing limitations like fast‐paced technological change outstripping checklist applicability, lack of formal update frameworks, absence of patient or stakeholder engagement, and limitations in generalizability across types of AI models or populations. Specifically, STARD‐AI was criticized for its very lengthy format and operational difficulty, while GAMER and ELEVATE‐GenAI raised concerns about applicability and exclusion of nongenerative systems. The comparison of 27 AI‐specific reporting checklists and guidelines against the AGREE II Tool indicated wide variation in quality across the six principal domains (D1–D6)—scope and purpose, stakeholder involvement, rigor of development, clarity of presentation, applicability, and editorial independence. The AASs were further examined to elucidate the visibility and influence of each checklist in the scientific community and in the public domain, with values between 0 and 288.

While AGREE II must be evaluated by a minimum of two authors, in our study, four authors evaluated the methodological rigor of the checklist using these domains. It was observed that the ICC for each domain measured by all four authors was >0.88, suggesting perfect agreement consistency between the authors.

**Figure 9:**
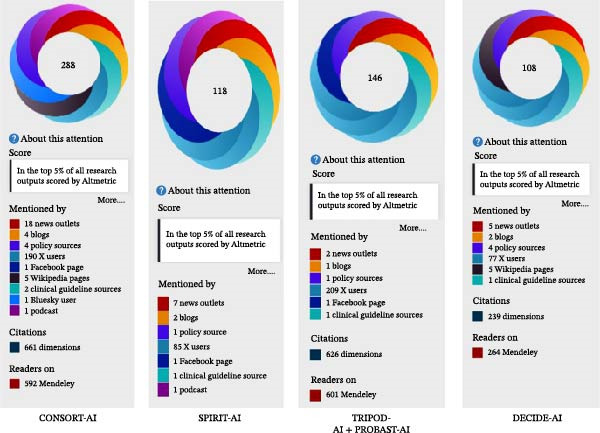
Altmetric Attention Score of the top four statements.

Among the first and most impactful instruments, CONSORT‐AI (AAS 288) (Figure 9) and SPIRIT‐AI (AAS 118) performed consistently at high levels in all six areas and produced almost perfect ratings (≥97%) on scope, rigor, and applicability.

Newer tools like PROBAST+AI (AAS 81), CHEERS‐AI (AAS 31), TRIPOD‐LLM (AAS 53), and STARD‐AI (AAS 33) illustrated equally high AGREE II scores across categories, indicating robust methodological design, cross‐disciplinary interaction, and reporting structure clarity. Conversely, multiple checklists like MINIMAR (AAS 23), clinical AI research (CAIR) (AAS 3), STAR‐ML (AAS 4), STAGER (AAS 2), MI‐CLEAR‐LLM (AAS 0), and ELSI (AAS 1) scored significantly lower, especially in rigor of development (D3) and applicability (D5).

This reflects shortcomings in systematic review of evidence, stakeholder involvement, and implementation support, thereby diminishing their practical application. For instance, MINIMAR received 18.23% in rigor and 27.78% in applicability, and this indicated insufficient detailed methodology and future updating procedures. Similarly, it was noticed that STAGER and ELSI both had low scores in editorial independence and stakeholder participation. Of the mid‐performing frameworks, APPRAISE‐AI (AAS 15) and CLEAR Derm (AAS 40) scored moderately to highly on scope and stakeholder involvement but had weaker scores in terms of applicability (34.72%–62.50%).

By contrast, newer frameworks like CHART (AAS 37), CLIX‐M (AAS 2), GAMER (AAS 12), and Clin‐STAR (AAS 4) were highly rated across most of the domains (≥80%), indicating robust methodological foundation and flexibility to the changing context of explainable and generative AI systems. Specifically, CHART was rated over 80% in all domains, indicating evenly strong methodological rigor, clarity, and representation of stakeholders.

For instance, CONSORT‐AI and SPIRIT‐AI, with the largest AAS, also demonstrated stronger domain performance, whereas lower AAS tools such as STAGER and MI‐CLEAR‐LLM indicated poorer methodological rigor. Still, exceptions like CLEAR (AAS 1) and CHEERS‐AI (AAS 31). Generally speaking, the AGREE II assessment shows that most AI reporting structures created after 2020 have high clarity and scope but mixed rigor and stakeholder involvement.

Although newer checklists like GAMER, PROBAST+AI, and CHEERS‐AI demonstrate almost optimal completeness, some of the initial or domain‐specific guidelines lack standardized update processes and actionable implementation plans. These results emphasize the need for evidence‐based, harmonized updates and increased expert participation to facilitate that AI reporting guidelines continue to be methodologically sound, transparent, and responsive to fast‐changing health research technologies.

The AGREE II assessment revealed a strong stratification of methodological rigor among the included AI reporting checklists. In general, Domains 1 (scope and purpose) and 4 (clarity of presentation) revealed high mean scores for most of the well‐established guidelines, while Domain 2 (stakeholder involvement), Domain 5 (applicability), and Domain 6 (editorial independence) revealed more variability.

The mean distribution of the domain scores were assessed for all the 27 checklists (Table 4). Among the top‐scoring guidelines, CHEERS‐AI revealed perfect or near‐perfect mean scores for all domains (D1 = 21.00 ± 0.00; D2 = 21.00 ± 0.00; D3 = 56.00 ± 0.00; D4 = 21.00 ± 0.00; D5 = 21.00 ± 0.00; D6 = 21.00 ± 0.00), suggesting outstanding methodological rigor, multidisciplinary stakeholder engagement, and complete editorial transparency. Likewise, CREMLS and PROBAST+AI revealed high scores for all six domains.

**Table 4:** Mean distribution of the domain scores.

AI‐specific reporting guidelines/checklist in healthcare	D1		D2		D3		D4		D5		D6	
	Mean	SD	Mean	SD	Mean	SD	Mean	SD	Mean	SD	Mean	SD
CHART	20.5	0.58	20.5	0.58	50.75	0.50	17.75	0.50	21	0.00	20.50	0.58
CLIX‐M	20.75	0.50	16.75	1.50	47.25	0.50	15.75	0.50	21	0.00	19.00	0.00
iCARE	19.75	1.50	14.75	0.50	20.25	1.89	7.5	0.58	16.75	0.50	21.00	0.00
Statistical Round of the Korean Journal of Anesthesiology	21	0.00	13.25	0.96	45.75	0.50	17.75	0.50	21	0.00	19.00	0.00
Clin‐STAR	21	0.00	13.5	1.73	54.00	2.31	19.5	1.73	21	0.00	21.00	0.00
TRIPOD‐LLM	20.5	0.58	21	0.00	55.00	1.15	21	0.00	21	0.00	15.00	0.00
STARD‐AI	21	0.00	21	0.00	55.50	0.58	21	0.00	21	0.00	15.00	0.00
GAMER	21	0.00	20.5	0.58	55.00	1.15	21	0.00	21	0.00	21.00	0.00
CLEAR	21	0.00	20	1.15	55.50	0.58	21	0.00	21	0.00	21.00	0.00
SPIRIT‐AI	20.5	0.58	21	0.00	55.00	1.15	21	0.00	21	0.00	21.00	0.00
CONSORT‐AI	21	0.00	21	0.00	54.75	1.50	21	0.00	21	0.00	21.00	0.00
CHEERS‐AI	21	0.00	21	0.00	56.00	0.00	21	0.00	21	0.00	21.00	0.00
CLAIM	21	0.00	9	0.00	22.00	0.82	11.75	0.50	14.25	0.50	14.25	0.50
STAR‐ML	19	0.00	15.75	0.50	32.25	0.96	16.5	1.00	18.5	1.00	9.75	0.50
TRIPOD‐AI + PROBAST‐AI	19	0.00	18.75	0.50	55.50	0.58	21	0.00	21	0.00	21.00	0.00
PROBAST+AI	21	0.00	21	0.00	55.00	1.15	21	0.00	21	0.00	21.00	0.00
CREMLS	20.5	0.58	21	0.00	56.00	0.00	21	0.00	21	0.00	21.00	0.00
DECIDE‐AI	21	0.00	21	0.00	56.00	0.00	21	0.00	21	0.00	15.50	0.58
MI‐CLEAR‐LLM	16.25	1.26	9.75	0.50	26.75	0.50	7.75	1.26	11.25	0.50	14.75	0.50
ELSI	17.5	1.73	12	0.00	20.00	2.00	12	0.00	12	0.00	10.00	0.82
CLEAR Derm Consensus Guidelines	21	0.00	16.5	1.00	43.25	2.87	17	0.00	14.25	0.50	16.75	0.50
ELEVATE‐GenAI	16	0.82	12.75	0.50	40.75	1.89	12	0.00	15.75	0.50	15.00	0.00
METRICS	17.75	0.50	16.25	0.50	41.50	1.73	8.25	1.50	14.25	0.50	16.75	0.50
Clinical AI Research (CAIR) checklist	17.75	0.50	10.75	0.50	28.50	1.00	12	0.00	16.5	1.00	9.75	0.50
STAGER checklist	17.75	0.96	13.5	0.58	27.75	0.50	10	0.82	10.25	0.50	3.00	0.00
MINIMAR	14.75	0.50	12	0.00	16.75	1.50	9.25	0.50	8	0.00	16.75	0.50
APPRAISE AI	14.75	0.50	17	0.00	50.50	0.58	9.25	0.50	15.75	1.26	19.00	0.00

CONSORT‐AI and SPIRIT‐AI also performed well, with maximum clarity (D4 = 21.00 ± 0.00) and applicability (D5 = 21.00 ± 0.00), along with strong development rigor (D3 ≈ 54.75−55.00). STARD‐AI and TRIPOD‐LLM performed well in terms of methodological rigor (D3 = 55.50 ± 0.58 and 55.00 ± 1.15, respectively) and clarity; however, both had relatively lower editorial independence (D6 ≈ 15.00), indicating a partial influence of funding or competing interests.

Moderately performing tools included CLEAR and GAMER, which scored well in terms of scope (D1 = 21.00 ± 0.00) and clarity (D4 = 21.00 ± 0.00) with high development rigor (D3 ≈ 55.00−55.50), although stakeholder involvement was slightly lower (D2 ≈ 20.00–20.50). DECIDE‐AI demonstrated excellent rigor (D3 = 56.00 ± 0.00) and clarity but reduced editorial independence (D6 = 15.50 ± 0.58). TRIPOD‐AI + PROBAST‐AI also showed strong rigor (D3 = 55.50 ± 0.58) with slightly lower stakeholder involvement (D2 = 18.75 ± 0.50).

Conversely, the earlier or independently developed checklists had relatively lower domain scores. CLAIM had very low stakeholder involvement (D2 = 9.00 ± 0.00) and moderate rigor (D3 = 22.00 ± 0.82), although high scope definition (D1 = 21.00 ± 0.00). MI‐CLEAR‐LLM had very low scores in all domains, especially in clarity (D4 = 7.75 ± 1.26) and applicability (D5 = 11.25 ± 0.50), suggesting very low structural refinement.

Likewise, the STAGER checklist and the CAIR checklist had moderate rigor (D3 ≈ 27.75−28.50) and low editorial independence (D6 ≈ 3.00−9.75), suggesting a lack of transparency in funding disclosure. MINIMAR had relatively low rigor (D3 = 16.75 ± 1.50) and applicability (D5 = 8.00 ± 0.00), suggesting low implementation guidance.

APPRAISE‐AI had an interesting set of scores, with high rigor of development (D3 = 50.50 ± 0.58) and high editorial independence (D6 = 19.00 ± 0.00), but relatively low clarity (D4 = 9.25 ± 0.50), suggesting a possible complexity in presentation despite high methodological quality. Domain‐wise analysis showed that Domain 3 (rigor of development) was the domain that most clearly distinguished high‐quality EQUATOR‐aligned AI extensions from new frameworks. Domain 2 (stakeholder involvement) and Domain 6 (editorial independence) were the domains that were most variable from one extension to another, often suggesting underreporting of multidisciplinary involvement or funding. Domain 4 (clarity of presentation) was the most consistent domain for high‐quality reporting extensions, suggesting well‐organized checklist design.

Pearson’s correlation test was used to determine the relationship between the AAS and the performance of the AGREE II domains among the 27 included guidelines. The findings showed a positive correlation for all six domains, suggesting that higher methodological quality was associated with increased online attention; although, the strength and significance of the correlation varied among the domains. There was a statistically significant moderate positive correlation between AAS and Domain 2 (stakeholder involvement) (*r* = 0.499, *p* = 0.008), suggesting that guidelines developed with more multidisciplinary input and better documentation of stakeholder involvement were associated with increased online attention. Similarly, Domain 4 (clarity of presentation) had a significant moderate positive correlation with AAS (*r* = 0.475, *p* = 0.012), suggesting that guidelines that were well‐organized and presented were associated with increased online dissemination and attention. Domain 3 (rigor of development) also showed a positive correlation with AAS (*r* = 0.423, *p* = 0.028), suggesting that guidelines that were methodologically sound and well‐developed were associated with increased attention scores. Although positive trends were found for Domain 5 (applicability) (*r* = 0.362, *p* = 0.064), Domain 6 (editorial independence) (*r* = 0.322, *p* = 0.101), and Domain 1 (scope and purpose) (*r* = 0.270, *p* = 0.173), none of these correlations were statistically significant at the 0.05 level. This suggests that although a better scope definition, implementation guidance, and editorial independence could be a factor in gaining more attention, the correlations were not as strong and may not reflect a consistent trend in this sample (Table 5).

**Table 5:** Correlation between Altmetric Attention Score and domains.

		D1	D2	D3	D4	D5	D6
Altmetric Attention Score	Pearson correlation	0.270	0.499	0.423	0.475	0.362	0.322
	*p* value	0.173	0.008	0.028	0.012	0.064	0.101
	*N*	27	27	27	27	27	27

## 4. Discussion

The findings of this review indicate a rapidly expanding yet uneven landscape of reporting standards for AI research in healthcare. The evaluation of co‐authorship and reporting guidelines in AI research across various databases reveals significant trends and developments in the field and clear growth in the number, scope, and specialization of these reporting guidelines; however, their uptake remains highly variable in academic literature and within digital dissemination metrics (Altmetric).

The analysis utilized data from Scopus, Dimensions AI Database, and PubMed, focusing on co‐authorship patterns, the emergence of new reporting guidelines, and the overall impact of these guidelines on AI‐related literature. The Scopus database identified 33 countries, with 23 demonstrating notable co‐authorship link strengths. Countries such as India, Norway, Greece, Slovenia, and Spain exhibited a rise in co‐authored literature post‐2023, indicating an increasing collaborative effort in AI research. Overlay visualizations highlighted these trends, suggesting a growing international interest in AI. In the Dimensions AI Database, a total of 406 authored documents were analyzed, with 62 meeting the criteria of at least two documents and two citations per author. Liu Xioxuan’s work on reporting guidelines for clinical trials involving AI was particularly prominent, with 11 documents and 1322 citations, showcasing the influence of this research on the field. The literature emphasized the importance of structured reporting in AI interventions, although it faced challenges due to the timing of its publication amidst a limited number of AI trials.

The systematic review identified 27 AI‐specific reporting guidelines and checklists published between 2020 and 2025, reflecting a global effort to enhance transparency and quality in AI‐derived health research. These guidelines varied in scope, addressing areas such as diagnostic accuracy, prediction modeling, and ethical use of AI. Notable early frameworks included SPIRIT‐AI and CONSORT‐AI, which adapted existing clinical trial frameworks to incorporate AI interventions. However, these guidelines faced limitations due to their timing and the evolving nature of AI terminology.

A central observation from this study is that foundational guidelines designed for broadly defined clinical AI interventions, such as SPIRIT‐AI [17] and CONSORT‐AI [18], consistently demonstrated higher bibliometric and Altmetric presence than more specialized or newly developed checklists. This outcome may partly reflect the influence of established parent frameworks—SPIRIT and CONSORT—which are long recognized and endorsed in traditional clinical trial research. Their AI‐specific adaptations thus benefit from inherent trust, existing publisher policies, and widespread familiarity among researchers. Furthermore, their emphasis on clinical trials aligns with regulatory and methodological priorities in AI evaluation, giving them a level of authority and utility that naturally encourages adoption.

Similarly, general‐purpose frameworks such as CLAIM (20), CAIR [20], and MINIMAR [21] showed robust visibility, indicating a strong early demand for baseline standardization around AI study reporting. These guidelines emerged at a time when concerns about reproducibility, methodological transparency, dataset bias, and overclaiming were already prominent in clinical AI discourse. Therefore, their early introduction positioned them as essential resources for researchers navigating a relatively unregulated and heterogeneous methodological environment. Early‐stage recognition also influenced social media–driven dissemination, with these guidelines receiving notable attention on platforms commonly monitored by Altmetric aggregators.

In contrast, more specialized guidelines—particularly those targeting narrow modalities or emerging AI architectures—demonstrated comparatively lower uptake. Examples include CLEAR‐Derm for dermatology imaging [23], CLEAR for radiomics [26], iCARE for interventional radiology [36], and CLIX‐M for explainable AI in decision support systems [35]. Their more limited bibliometric footprint may be understood through several lenses. First, such tools tend to address smaller professional communities, inherently limiting their potential citation pool. Second, niche guidelines are often adopted primarily when a field reaches a methodological maturity that demands standardization; until then, usage remains inconsistent. Third, clinical subspecialties vary widely in AI readiness, regulatory expectations, and methodological culture. As a result, reporting norms evolve heterogeneously across disciplines.

Generative AI imposes additional complexity. Recent checklists such as MI‐CLEAR‐LLM (31), METRICS for generative AI in education and practice [32], STAGER for evaluating generative AI reliability [33], TRIPOD‐LLM for predictive model studies using LLMs [39], and GAMER for the use of generative AI tools in medical research [41] exhibited sharp increases in Altmetric activity but comparatively fewer traditional citations. This divergence suggests that generative AI guidelines are driven primarily by rapid social and technological currents rather than by established academic adoption cycles. The prominence of LLM‐related guidelines on social platforms reflects broader societal interest, faster community discourse, and increasing scrutiny about reliability, hallucinations, ethics, and safety of generative models. However, the slower accumulation of citations likely reflects a lag in the production of mature, peer‐reviewed generative AI studies adhering to these recommendations. The speed of generative AI development also challenges the traditional scholarly publishing process, creating an ongoing tension between relevance and formal uptake.

Several guidelines emerging after 2023—including CHEERS‐AI for economic evaluations (28), APPRAISE‐AI [25], CREMLS [29], CHART for chatbot studies [34], ELEVATE‐GenAI for health economics and outcomes research [43], and PROBAST+AI for risk of bias appraisal [42]—revealed modest but growing visibility. Many of these represent extensions of longstanding methodological standards (e.g., CHEERS, PROBAST) into AI contexts, suggesting that established methodological frameworks continue to anchor credibility and shape research expectations. Their emerging adoption patterns indicate that the field is shifting beyond basic reporting adequacy toward evaluating cost‐effectiveness, safety, applicability, trustworthiness, and bias—domains that reflect a maturation of AI integration into healthcare systems.

Recent frameworks like STAR‐ML, APPRAISE‐AI, and CHEERS‐AI aimed to address these limitations but were criticized for issues such as low procedural transparency and limited stakeholder engagement. Newer tools introduced in 2025, including CHART, CLIX‐M, and GAMER, represent an evolution in addressing the challenges posed by generative AI and multimodal models. Despite their advancements, these tools still grapple with rapid technological changes and the need for formal update frameworks. The comparison of the 27 reporting checklists against the AGREE II Tool revealed significant variations in quality across six principal domains: scope and purpose, stakeholder involvement, rigor of development, clarity of presentation, applicability, and editorial independence. CONSORT‐AI and SPIRIT‐AI emerged as the most impactful instruments, scoring highly across all domains. In contrast, several checklists, such as MINIMAR and CAIR, received lower scores, particularly in rigor of development and applicability, indicating deficiencies in methodological rigor and stakeholder involvement. The AAS provided additional insights into the visibility and influence of each checklist within the scientific community. Higher AAS values were associated with guidelines that demonstrated robust methodological design and cross‐disciplinary interaction. For instance, CONSORT‐AI achieved an AAS of 288, reflecting its significant impact, while other frameworks like MINIMAR and ELSI scored much lower, highlighting the need for improvement in their development processes.

Guo et al. [3] conducted a bibliometric study which was done using the Web of Science database; the results showed a rapid rise in AI‐related health research around 2015, driven largely by advances in deep learning, increased computational capacity, and the digitalization of clinical data. The authors highlighted gaps such as the need for more interdisciplinary studies, standardized evaluation frameworks, and real‐world clinical validation. We found that our result was in the same consensus; however, we additionally found that the majority of the research regarding AI reporting guidelines was published between the years 2020 and 2025. Another bibliometric analysis was done by Tran et al. [4] mapped the global evolution of AI research in health and medicine from 1990 to 2018, the authors used Web of Science. The results indicated exponential growth, particularly in the last decade, driven by improved computational tools and broader adoption of digital technologies in health systems. Topol et al. [10] published a commentary about involving new guidelines for AI clinical research. The authors highlighted reporting frameworks, specifically CONSORT‐AI and SPIRIT‐AI, and included common issues such as algorithmic bias, lack of external testing, poor documentation of model development, and unrealistically optimistic performance claims.

Shelmerdine et al. [44] conducted a review of the reporting guidelines for AI in healthcare; the authors evaluated suitability, completeness, and alignment with the methodological demands of AI research. They noted that many traditional reporting standards insufficiently address AI‐specific concepts, leading to the development of newer extensions such as CONSORT‐AI and SPIRIT‐AI. These AI‐focused tools offer clearer expectations regarding data provenance, handling of bias, external validation, and deployment considerations. However, the authors also recognized persistent challenges, particularly the lack of consensus on ideal evaluation metrics, the complexity of evolving ML workflows, and limited guidance for unsupervised or continuously learning models. The review concludes that while recent guidelines represent meaningful progress, researchers must carefully select and apply the most appropriate framework, and further refinement is needed to ensure high‐quality, clinically meaningful AI research.

Another systematic review and meta‐analysis was performed by Kolbinger et al. [45] for quality in medical AI studies across multiple disciplines. TRIPOD, CONSORT‐AI, SPIRIT‐AI, and CLAIM reporting guidelines were evaluated. This review found evidence of selective reporting, where performance metrics were highlighted, but methodological limitations were omitted. The authors of the review recommend stronger journal enforcement of guidelines, improved reviewer training, and the development of unified, domain‐specific tools that reduce redundancy. Klontzas et al. [46] provided a practical guide for researchers navigating the growing number of AI reporting guidelines. CLAIM, TRIPOD‐AI, CONSORT‐AI, and SPIRIT‐AI were assessed, in which the authors highlighted the importance of describing dataset formation, model architecture, training methodology, evaluation metrics, uncertainty estimates, and human–AI interaction.

The key reason identified for poor stakeholder requirements was in the fact that many AI reporting guidelines have their roots in computational or engineering research groups, where the development is primarily led by data scientists and methodologists. In contrast to traditional clinical guideline development, which usually requires the involvement of clinicians, statisticians, methodologists, and patient representatives, AI frameworks may not have formalized multidisciplinary groups.

## 5. Strengths and Limitations

This study presents a comprehensive evaluation of AI‐specific reporting guidelines through a multimethod approach, yet several limitations must be acknowledged when interpreting the findings.

### 5.1. Strengths

1. Comprehensive multimethod design: This review synthesizes findings from three complementary methodologies: a systematic review, a bibliometric analysis, and an Altmetric analysis. This tripartite approach provides a holistic view, assessing not only the intrinsic quality and content of the guidelines but also their scholarly footprint, collaborative networks, and broader dissemination impact, offering insights beyond what any single method could achieve.
2. Rigorous and standardized quality appraisal: The methodological quality of the included guidelines was critically appraised using the AGREE II instrument, a validated and internationally recognized gold standard for guideline assessment. The independent evaluation by multiple reviewers minimized bias and ensured a robust, transparent scoring process across six key domains of guideline development.
3. Systematic and reproducible methodology: The systematic review component adhered strictly to the PRISMA 2020 statement, with a protocol prospectively registered in PROSPERO. The employment of a dual‐independent reviewer system for study selection and data extraction, alongside detailed reporting of search strategies across multiple databases, ensures high methodological rigor and reproducibility.
4. Novel and timely contribution: This study addresses a critical and rapidly evolving gap in the literature. By focusing exclusively on AI‐specific reporting instruments (2020–2025), it provides a novel, much‐needed synoptic appraisal of a foundational yet fragmented ecosystem, capturing the field’s evolution from early general frameworks to specialized tools for generative AI and LLMs.
5. Identification of actionable gaps: The analysis moves beyond mere description to identify specific, actionable weaknesses within the guideline landscape. By pinpointing common deficiencies—such as the lack of formal update procedures, insufficient stakeholder and patient involvement, and weak applicability domains—the study provides clear, evidence‐based directions for future guideline development and refinement.

## 6. Limitations

1. Database and indexing biases: The findings are constrained by the coverage and indexing algorithms of the selected databases (Scopus, PubMed, and Dimensions AI). Relevant guidelines published in non‐indexed journals, repositories, or in languages other than English may have been omitted. Similarly, Altmetric data can be skewed by the popularity of certain social media platforms and may not fully capture all forms of scholarly or public engagement.
2. Inherent time‐lag bias: A significant interpretive challenge is the varying age of the included guidelines. Foundational tools published in 2020−2021 (e.g., CONSORT‐AI, SPIRIT‐AI) have had substantially more time to accumulate citations, Altmetric attention, and demonstrate adoption than those published in 2024−2025. This temporal disparity can skew impact assessments, potentially undervaluing the methodological robustness of newer frameworks designed for emerging technologies.
3. Assessment of guideline reporting vs. adherence: This review appraises the guidelines as published documents and their declared impact metrics. It does not evaluate the actual adherence to these guidelines within the broader corpus of AI health research literature. A guideline can be of high methodological quality yet see poor implementation, a critical dimension of effectiveness not captured here.
4. Language and access bias: The restriction to English‐language, open‐access publications, while pragmatic, may have excluded relevant guidelines published in other languages or behind paywalls. This limits the global representativeness of the findings and may underrepresent guidelines developed within specific regional or linguistic contexts.
5. Heterogeneity of guidelines: The included instruments vary considerably in scope, target audience, and intended use (e.g., clinical trials vs. diagnostic accuracy vs. economic evaluations). While the AGREE II Tool allows for comparative quality assessment, direct comparisons of impact scores between such fundamentally different tools must be interpreted with caution, as they serve distinct niches within the research ecosystem.
6. Subjectivity in quality appraisal: Although the AGREE II Tool provides a standardized framework, the scoring process inherently involves a degree of expert judgment, particularly for domains like “Applicability” and “Editorial Independence.” While mitigated by using multiple independent reviewers, some residual subjectivity is unavoidable.
7. Additional to the above, it was observed that there were core structural limitations in the guidelines, including: fragmented ecosystem: AI guidelines tend to be domain‐specific (e.g., imaging, prediction models, and generative AI), resulting in parallel developments rather than a common framework. Interdisciplinary gaps: AI research is inherently an intersection of medicine, statistics, and computer science. Institutional silos may hinder the complete integration of the three disciplines during guideline development. Limited implementation science integration: Some frameworks have a lower score in applicability because they do not include criteria for auditing, resource issues, and implementation strategies—indicating the limited integration of implementation science principles. Editorial independence reporting: Many new AI guidelines have limited disclosure of funding sources and conflict of interest statements, possibly because of smaller groups of developers or industry collaboration dynamics. Absence of mandatory adoption mechanisms: Unlike CONSORT extensions, which are generally accepted by journals, many AI guidelines do not have a formal adoption process, thereby reducing the need for rigorous consensus development.

Taken together, these strengths underscore the validity and novelty of our findings, while the limitations contextualize their scope. Future research would benefit from longitudinal tracking of guideline adherence, inclusion of non‐English literature, and qualitative assessments of developer and end‐user experiences to build upon this foundational mapping of the field.

## 7. Conclusion

The findings underscore the importance of developing evidence‐based, harmonized updates to AI reporting guidelines to ensure they remain methodologically sound and relevant in the face of rapid technological advancements. Increased expert participation and stakeholder engagement are crucial for enhancing the applicability and rigor of these guidelines. As the landscape of AI research continues to evolve, it is essential for reporting frameworks to adapt accordingly, ensuring that they effectively address the complexities and challenges associated with AI in health research.

In summary, the analysis of co‐authorship trends and reporting guidelines in AI research reveals a dynamic and rapidly evolving field. While significant progress has been made in establishing frameworks for reporting AI interventions, ongoing efforts are needed to enhance their quality, applicability, and responsiveness to the changing landscape of health research technologies.

## Funding

The research has not received any specific funding.

## Disclosure

This review is a part of the Ph.D. dissertation being carried out in the Department of Public Health Dentistry at Manipal College of Dental Sciences, Mangalore, Manipal Academy of Higher Education, Karnataka, Manipal, 576104, India. This review was presented as a paper at the 29th Indian Association of Public Health Dentistry National Conference held in Mangalore from 28th to 30th November 2025.

## Conflicts of Interest

The authors declare no conflicts of interest.

## Supporting Information

Additional supporting information can be found online in the Supporting Information section.

## Supporting information


**Supporting Information 1** PRISMA 2020 Checklist.

**Supporting Information 2** Bibliometric reviews of the biomedical literature (BIBLIO) checklist.

## Data Availability

Data sharing is not applicable to this article, as no datasets were generated or analyzed during the current study.
